# Correlation of CD24 expression with histological grading and TNM staging of retinoblastoma

**DOI:** 10.12669/pjms.321.8828

**Published:** 2016

**Authors:** Syed Muhammad Ishaq, Shahnaz Imdad Kehar, Shahid Zafar, Syed Furqan Ul Hasan

**Affiliations:** 1 Dr. Syed Muhammad Ishaque, MBBS, M.Phil, Consultant Pathologist, Bolan Medical Complex & Sandeman Provincial Hospital, Quetta. Pakistan; 2 Dr. Shahnaz Imdad Kehar, MBBS, M.Phil, Associate Professor, Department of Pathology, BMSI, JPMC, Karachi, Pakistan; 3 Dr. Shahid Zafar, MBBS, M.Phil, Assistant Professor, Department of Pathology, Liaquat College of Medicines & Dentistry, Karachi, Pakistan; 4 Dr. Syed Furqual Ul Hazan, MBBS, M.Phil, Associate Professor, NICH, Karachi, Pakistan

**Keywords:** Retinoblastoma, Cluster of Differentiation (CD) 24, Histological grade, TNM stage

## Abstract

**Objective:**

Correlation of CD24 expression with histological grading and TNM staging of retinoblastoma.

**Methods:**

This cross-sectional study was conducted in the Department of Pathology, BMSI, JPMC and NICH from 1^st^ January 2009 to 31^st^ December 2013. A total 68 diagnosed cases of retinoblastoma were selected for CD24 immuno staining. The data was analyzed by using SPSS version 22.

**Results:**

Out of 68 cases 7.35% showed grade 1 followed by 11.76% in G2, 26.47% in G3 and 54.41% in G4. Majority of cases i.e. 60.29% in stage IV followed by 19.11% in stage I, 10.29% each in stage II and stage III. CD24 immuno staining positivity was seen in majority of grade 3 and grade 4. In grade 3, 38.88% showed moderate and 22.22% strong immuno reaction. Amongst grade 4, 40.54% showed moderate and 13.51% strong positive. Variable immuno pattern was observed according to TNM staging. In stage I, 46.15% showed moderate and 7.69% strong positivity, while in stage II, 57.14% were negative for saining. In stage III, 42.85% were negative while 28.57% each showed moderate and strong staining. Majority of cases in stage IV i.e. 48.78% were negative for staining while 34.14%, 17.07% showed moderate and severe CD24 immuno staining.

**Conclusion:**

Majority of grade I retinoblastoma were in TNM stage I & II and mostly were immuno negative. Lymph node and distant metastatic cases were 75% in G4 and 25% in G3, all of them showed moderate to strong immunoreactivity. These results showed that CD24 expression may be a marker of poor prognosis in retinoblastoma. Whereas TNM staging of retinoblastomas with CD24 expression had varying pattern and showed no significant correlation between them.

## INTRODUCTION

Retinoblastoma is a highly malignant tumor, originating from primitive and immature cells of neural retina and is considered the most common intraocular primary childhood malignancy.^1^-^4^

Worldwide 5,000 new cases of retinoblastoma are diagnosed annually. Incidence ranges from 1 in 15,000 to 1 in 18,000 live birth and accounts for 2 to 4% of all childhood malignant neoplasm.^5^,^6^ In US its incidence is 11.8 per million under 4 years of age.^6^ African countries showed higher incidence of retinoblastoma accounting for 10 to15%. North America, Australia and Asian sub continent accounts for 2 to 4% of neoplasm in children.^7^-^9^ According to Shaukat Khanum^10^ collective Cancer Registry, (1994 to 2011) retinoblastoma accounts 6^th^ most common malignancy with 5.09% frequency. Retinoblastoma is seen both as hereditary and non hereditary form.^11^-^14^ The tumor with choroid, optic nerve and orbital invasion has a high risk of extra ocular relapse.^15^-^19^

CD24 is a prognostic marker, used for hematopoietic and neuronal cell differentiation, involved in cell repair, proliferation, apoptosis and cell adhesion control.^15^ Functionally CD24 is an alternative ligand of p-selectin, an adhesion receptor expressed on activated endothelial cells and platelets.^20^ It is a small protein containing 27 amino acid, glycosylated and phosphatidylinostiol anchor which binds to membrane.^21^ It is a specific B cell marker expressed at the early stage of B cell development. High level of CD24 expression are associated with aggressive course, poor prognosis of the disease and short patient survival times.^22^,^23^. CD24 over expression is seen in hematological B cell tumor as well as non haematopoeitic tumors and its expression may contribute to metastasis, poor prognosis and is significantly associated with histological grades of tumor.^24^ Positive correlation between CD24 and pathological cancer grade (Low and high) shows intensity of malignancy.^15^,^20^

Present study was designed to evaluate the differential expression of CD24 in different histological grades of retinoblastoma and to assess the prognostic importance of CD24 expression by correlation with TNM staging and histological grading.

## METHODS

The study was performed at the department of Pathology Basic Medical Sciences Institute, Jinnah Postgraduate Medical Center with collaboration of National Institute of Child Health Karachi. From 1^st^ January 2009 to 31^st^ December 2013. A total of 68 cases of retinoblastoma were included in this study. These patients were operated at Ophthalmology department of JPMC Karachi. Poorly fixed & inadequate tissue, ocular tumor other than retinoblastoma and metastatic tumors were excluded. Goat CD24 (c-20); sc-7034, polyclonal antibody in concentrated form were diluted 1:25 with Phosphate Buffered Saline and Horseradish Peroxidase, Goat anti horse, sc-2448 (secondary kit) in concentrated form were diluted 1:200 with Phosphate Buffered Saline boths were from SANTA CRUZ BIOTECNOLOGY were used. Formalin fixed, paraffin embedded blocks, surgical pathology, clinical records, Hemtoxylin & Eosin slides and approximately 5 microns section were cut on to poly-L-lysine coated slides for antibody were used. CD24 expression was expressed by observing the intensity and extent of immunostaining each graded on a scale of 1-3.

♦Extent of cells were as follows, 0= 100% cells were negative, 1+ = <10% cells were positive, 2+=10-50% cells were positive, 3+=>50% cells were positive.

*Intensities of reactivity as follows; 0= no staining, 1+= weak staining, 2+=moderate staining, 3+= strong staining.

Intensity and extent in each case were added and the additive scores were categorized as:^25^

Category I = 0 to 2 score (Immuno negative)

Category II = 3 to 4 score (Immuno positive)

Category III = 5 and 6 score (Immuno positive)

Human spleen known to be positive for CD24 was used as positive control. The relevant clinical information and others data were collected. All slides were studied under light microscope using scanner (4x), low power (10x) followed by high power (40x). The data was analyzed by using Statistical Package for Social Sciences (SPSS) version 22.

## RESULTS

**Fig.1:**
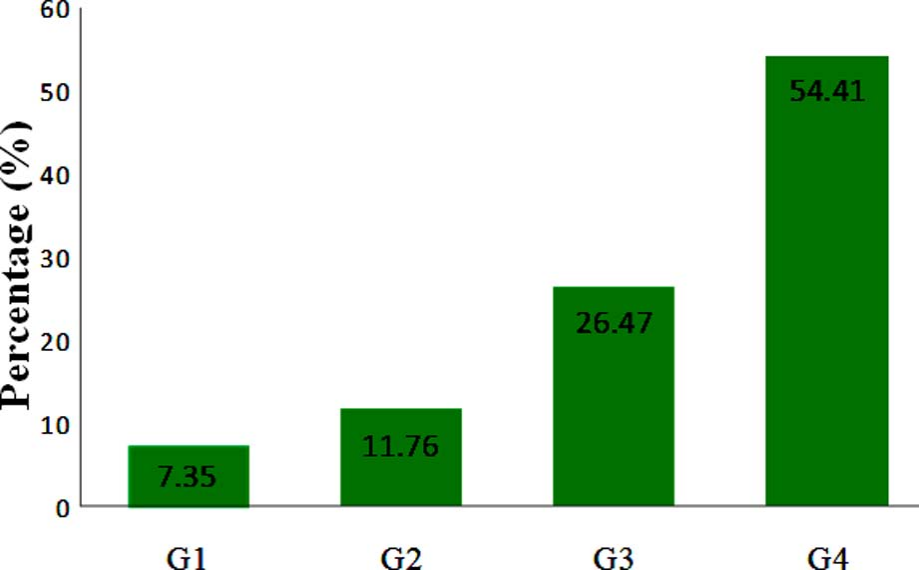
distribution of retinoblastoma cases according to histological grades (n=68).

Out of 68 cases, 54.41% were undifferentiated (G4), 26.47% were poorly differentiated (G3), 11.67% were moderately differentiated (G2), and 07.35% were well differentiated (G1) respectively (Fig.1).

Distribution of retinoblastoma according to TNM Staging system is shown in Table-I. Out of 68 selected cases majority i-e 60.29% were in Stage IV, 10.29% each were in Stage III (pT3, N0, M0) and Stage II (pT2, N0, M0), while 19.11% were in Stage I (pT1, N0, M0) respectively. Stage IV varied from pT4, N0, M0 to PT4, N1, M1, since 09.75% showed regional lymph nodes metastasis. While distant metastasis was seen in 07.31%.

**Table-I:** distribution of retinoblastoma according to TNM staging (n=68).

STAGE		No of cases		% of total cases	95% CI
Stage I (pT1,N0,M0)		13		19.11	11.06-29.75
Stage II (pT2,N0,M0)		07		10.29	4.61-19.3
Stage III (pT3, N0,M0)		07		10.29	4.61-19.3
Stage IV	(pT4,N0,M0)	34	41	60.29	48.34-71.4
	(pT4,N1,M1)	07			

Total	68	100			

CI: Confidence interval.

**Fig.2:**
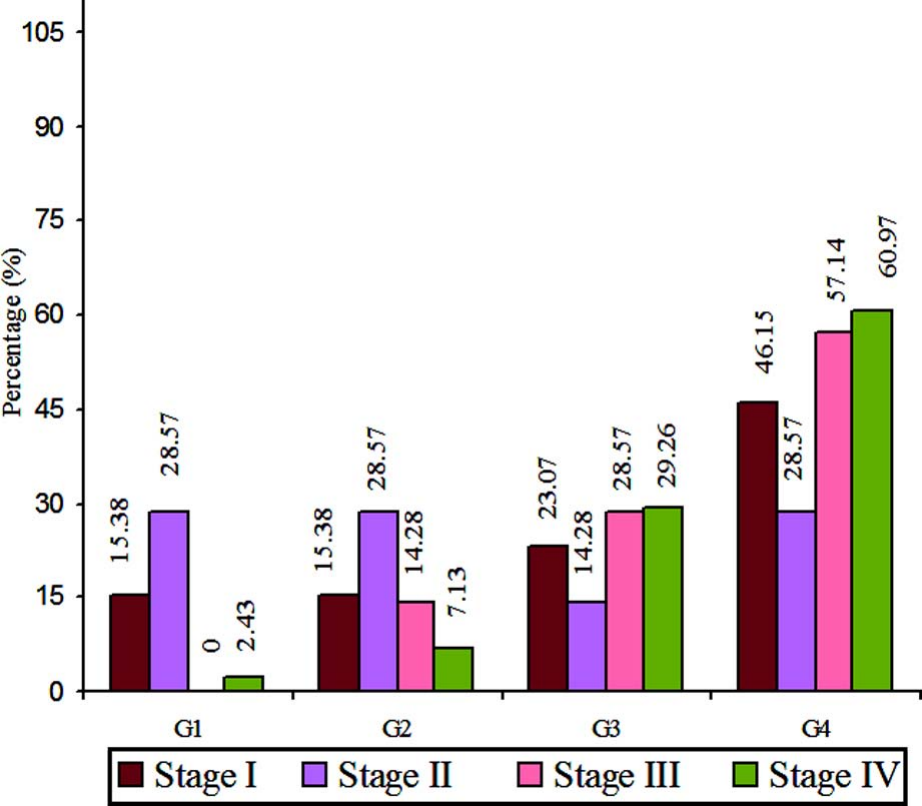
Correlation of retinoblastoma according to histopathological grades and TNM staging (n= 68).

On comparison and correlation of histopathological grades and TNM Stages. Out of 68 cases, 19.11% were in Stage I, out of these 15.38% each were in histological G1 and G2, 23.07% were in G3, while 46.15% were seen in G4. A total no of 10.29% were in Stage II showing 28.57% each in G1, G2 and G4, while 14.28% were in G3. Stage III shows 10.29% out of these 14.28% in G2, 28.57% in G3 and 57.14% were in G4. TNM Stage IV were seen in total no 60.29%, out of these 02.43% were in histopathological G1, 07.13 were in G2, and 29.26% were in G3, while 60.97% were seen in G4 (Fig.2).

Additive score for immunoreactivity according to histopathological grades are sown in Table-II. Out of 05 cases with grade 1 histology, 80% reveal category I staining. Out of 08 cases belonging to G2, 37.50% showed category II and category III staining. Out of 18 cases with histological G3, 61.11% showed either category II or category III staining. Amongst 37 cases in G4, 54.05% showed either category II or category III staining.

**Table-II:** CD24 immuno reactivity according to different histopathological grades of retinoblastoma (n = 68).

Grade of Retinoblastoma	Category I	Category II	Category III	Total
Grade 1	04 (80%)	01 (20%)	00	05
Grade 2	05 (62.50%)	02 (25%)	01 (12.50%)	08
Grade 3	07 (38.88%)	07 (38.88%)	04 (22.22%)	18
Grade 4	17 (45.94%)	15 (40.54%)	05 (13.51%)	37

Total	32 (47.05%)	26 (38.23%)	10 (14.70%)	68

P value = 0.24, Chi Square = 4.07

Additive score for immuno staining according to TNM Stage is shown in Table-III. Out of 68 cases, 46.15% showed category I additive score in stage I, 42.85% in stage II showed category II additive score and 57.14% in stage III showed either category II or category III additive score. In stage IV, 51.21% shows category II and category III additive score.

**Table-III:** CD24 immuno staining according to TNM staging of retinoblastoma (n=68).

TNM Stage	Category I	Category II	Category III	Total
Stage I	06 (46.15%)	06 (46.15%)	01 (07.69%)	13
Stage II	04 (57.14%)	03 (42.85%)	00	07
Stage III	03 (42.85%)	02 (28.57%)	02 (28.57%)	07
Stage IV	20 (48.78%)	14 (34.14)	07 (17.07%)	41

Total	33 (48.52%)	25 (36.76%)	10 (14.70%)	68

P value = 0.771, Chi Square = 3.29

In this study the regional lymph nodes metastasis was seen in 9.75% cases. Majority (75%) of these were in histopathological G4 and 25% in G3. All these cases with additive score of category II and III staining. We also found 07.31% cases with CNS metastasis, all were in histopathological G4, additive score of category II or III staining.

**Photomicrograph.1:**
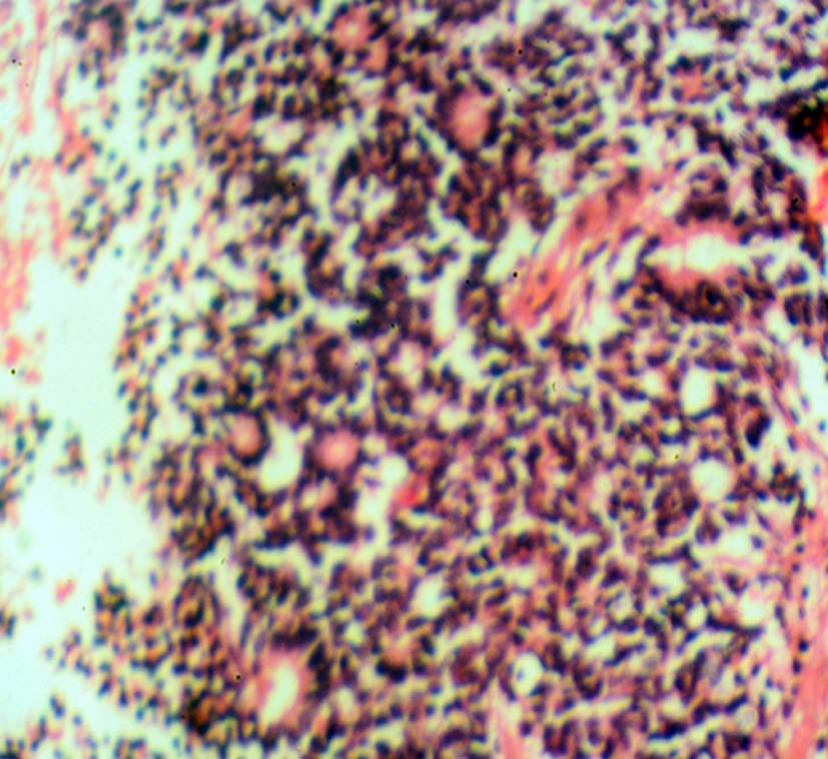
Well differentiated retinoblastoma, TNM Stage II, (H&E ×10)

**Photomicrograph.2:**
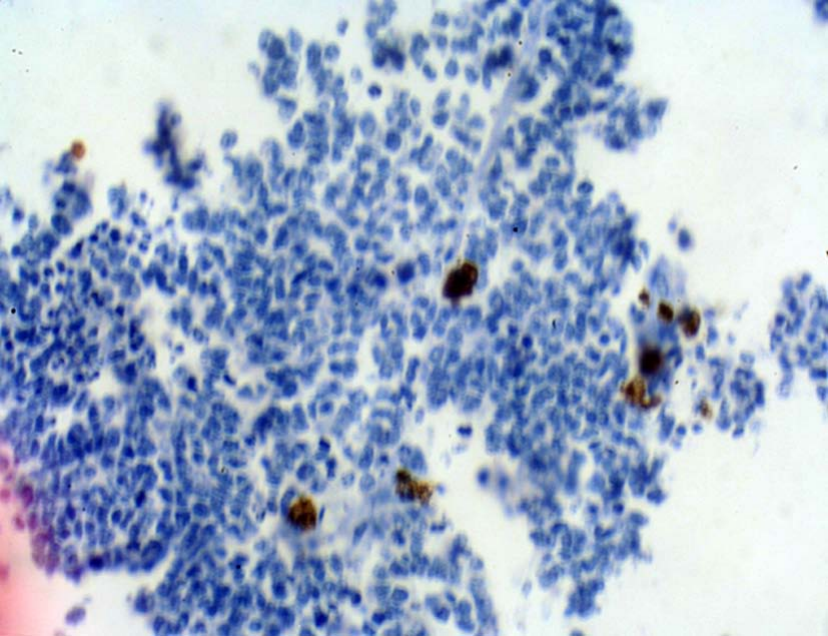
Poorly differentiated retinoblastoma, TNM Stage IV, showed moderate CD24 staining. (IHC ×40)

**Photomicrograph.3:**
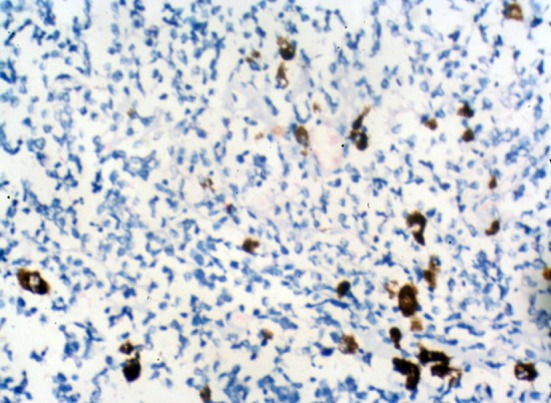
Undifferentiated retinoblastoma, TNM Stage II, showed moderate CD24 staining. (IHC ×40)

## DISCUSSION

In the present study varied histopathological grades were seen amongst the 68 cases 07.35%, 11.76%, 26.47% and 54.41% were in G1, G2, G3 and G4 respectively. Nigerian study by Owoeys et al.^11^ reported 17.4%, 00% and 82% in histopathological G1, G2 and G3 respectively, while Chinese study by Li et al.^15^ reported 24%, 14% and 62% in histopathological G1, G2 and G3 respectively. No case was reported in histopathological grade 4 by both of them. While Present study showing majority of cases in histopathological G4. TNM Staging in this series show 19.1%, 10.3%.10.3% and 60.3% in stage I, stage II, stage III and stage IV respectively. Thus majority of cases were in TNM stage IV.

This study showed category II and III immune reactivity for CD24 in majority of cases i.e. 61.11% with histopathological G3, 54.05% in G4. Whereas category I, considered as immuno negative was seen in 80% of in histopathological G1 and 62.50% cases in G2. These results are in accordance with Chinese study by Li et al.^15^ who reported CD24 immuno reaction positivity in 74.19% in histopathological G3, 57.14% in G2, while CD24 immuno negative in 41.66% in G1 and none of the case were reported in G4. These slight variations could be due to environmental, genetic differences and sample size variation. The present study thus shows an obvious correlation between CD24 immuno expression with increasing histological grade of retinoblastoma.

Although considerable number of G4 cases show CD24 expression yet the p value is not significant, which may be due to limitation of sample size in our study. This study also emphasizes that majority of well differentiated retinoblastoma were in lower TNM stage i-e stage I and II and a vast majority showed negative staining for CD24.

When CD24 immunoexpression was correlated with TNM staging, varying pattern was found in the present study. Although a considerable number of cases showed positivity for CD24 expression in all four TNM stages, it was observed that almost the similar number of cases in stages I, II, III and IV showed total negativity for CD24 expression. The p value for correlation between CD24 with TNM staging was not significant, thus no significant correlation between CD24 expression with TNM stage of the retinoblastoma. Karahan et al.^24^ also reported no correlation between CD24 expression and TNM stages in Colorectal Carcinoma.

The cases with lymph nodes and distant metastasis were mostly found in histopathological G4 and a single case in G3. All of these cases showed moderate to strong immuno reactivity for CD24. These results may be valuable in concluding that CD24 expression may be a marker of poor prognosis in retinoblastoma.

## CONCLUSION

Present study shows that CD24 expression progressively increased with higher histopathological grades, therefore a significant correlation was found between histological grade and CD24 expression. Maximum number of cases were in TNM stage IV and a considerable number of these cases showed positive immuno staining for CD24, however no significant correlation was seen between CD24 expression and TNM staging. Majority of cases with lymph nodes and distant metastasis were in histopathological G4 and showed CD24 immuno expression of moderate to strong intensity, thus proving that expression of CD24 may be a marker of poor prognosis.
